# Impact of the coronavirus disease 2019 epidemic on Taiwanese health care networks: Sharing experiences on a community hospital’s responses

**DOI:** 10.7189/jogh.10.020376

**Published:** 2020-12

**Authors:** Chia-Chi Yen, Shu-Ling Chain, Hsien-Ju Lee, Chih-Hao Chen, Min-Yi Lee, Shu-Yi Wei, Hsiu-Min Chen, Hsiao-Ling Cheng

**Affiliations:** 1 Superintendent’s Office, Kaohsiung Municipal Min-Sheng Hospital, Kaohsiung, Taiwan; 2 Department of Nutrition, Institute of Biomedical Nutrition, Hungkuang University, Taichung, Taiwan; 3 Department of Business Management, National Sun Yat-Sen University, Kaohsiung, Taiwan; 4 Department of Medical Education and Research Center, Kaohsiung Municipal Min-Sheng Hospital, Kaohsiung, Taiwan; 5 Department of Nursing, Kaohsiung Municipal Min-Sheng Hospital, Kaohsiung, Taiwan; 6 Division of Neurosurgery, Department of Surgery, Kaohsiung Municipal Min-Sheng Hospital, Kaohsiung, Taiwan; 7 Division of Cardiology, Department of Internal Medicine, Kaohsiung Municipal Min-Sheng Hospital, Kaohsiung, Taiwan; 8 Division of Nephrology, Department of Internal Medicine, Kaohsiung Municipal Min-Sheng Hospital, Kaohsiung, Taiwan.; 9 Department of Medical Education and Research, Kaohsiung Veterans General Hospital, Kaohsiung, Taiwan.; 10 Department of Pharmacy, Kaohsiung Municipal Min-Sheng Hospital, Kaohsiung, Taiwan

**Figure:**
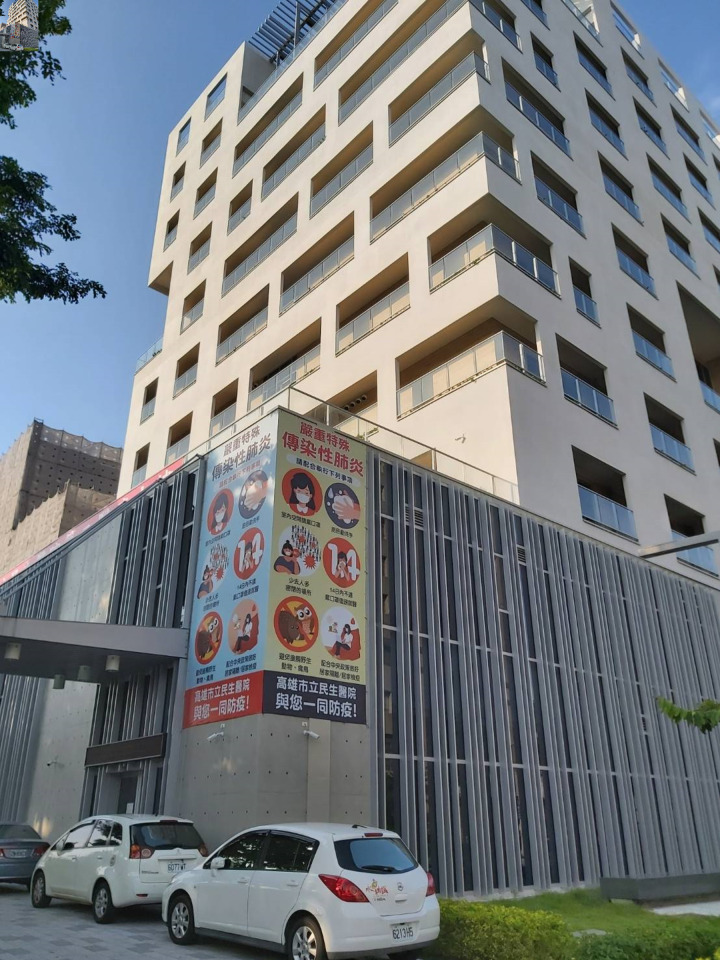
Photo: Second Medical Building, Kaohsiung Municipal Min-Sheng Hospital, Taiwan (August 2020). From the authors’ own collection, used with permission.

## TAIWANESE COMMUNITY HOSPITAL’S EPIDEMIC PREVENTION OUTCOMES

Taiwan’s Kaohsiung Municipal Min-Sheng Hospital is a community hospital with a total 350 beds and 644 staff and has been appointed as designated specialty hospital for health care network of infectious diseases prevention by the Department of Health, Kaohsiung City Government since the SARS event on 2003. For response to the coronavirus disease 2019 (COVID-19), the Ministry of Health and Welfare (MOHW) listed this hospital as one of 167 nationally appointed community testing hospitals, wherein the hospital took up the responsibility of screening and admitting patients diagnosed with the newly rising infectious disease.

COVID-19 epidemic broke out in December 2019. The virus has already spread internationally and alerted public health and health care systems. By 11 March 2020, the WHO had announced COVID-19 to be a pandemic. It indicates that enforcing appropriate emergency management mechanisms must be in time. In geographical location, Taiwan is a neighbour of Mainland China, moreover, people of both maintain frequent contact by which Taiwan was originally predicted to be one of the most severely impacted area by the epidemic when COVID-19 broke out [1]. However, Taiwan take rapid and proactive actions on the pandemic due to her SARS-fighting experiences on 2003.

As of 9 August 2020, the data of Total case/Total death per 1M population in countries those have similar GDP with Taiwan were Taiwan (20/0.3), Italy (4131/582), Republic of Korea (285/6), Spain (6723/610), Czechia (1686/36), Portugal (5136/171), and Greece (506/20) [2,3]. As a result, Taiwan’s epidemic prevention outcomes received unanimous recognition from foreign public health. Several countries are paying close attention to Taiwan’s successful experience in epidemic prevention.

Herein, as an example of community hospital’s emergency management mechanisms to the impact of COVID-19 epidemic, we shared those management mechanisms of Taiwan’s Kaohsiung Municipal Min-Sheng Hospital including a support system for all health care and administrative personnel within the community hospital as well as a treatment-seeking control system for members of the community.

## COVID-19 EPIDEMIC RESPONSE STRATEGIES

Five major STRATEGIES set up when the government publicised information related to the COVID-19 epidemic (**Table 1**).

**Table 1:** Summary of Min-Sheng hospital’s COVID-19 epidemic prevention strategies

Strategies		Outcome	
1.Entry and Exit Access Control	Body temperature screening		This strategy is usefully distinct from the high- and low-risk persons and prevents cross-infection among personnel and cuts off potential transmission routes.
	Questionnaire on travel history, occupation, contact history, cluster history		
	Inquires if have a respiratory tract disease		
	Requires to wear a mask		
2. Management of Wards	Subdividing hospital staff by limiting the job descriptions		This strategy prevented health care persons from contacting with confirmed cases, or would result in short staffing and affecting the capacity of health care.
	Fixed personnel work in fixed zones during fixed shift periods		
	Triaging patients visiting hospitals		
3. Management of Hospital staff	All staff on the roster lists		This strategy upheld the safety of health care persons and provided them with a safe environment would protect them from the risk.
	Distributed masks for staff, installed more hand-washing devices, and increased the sanitisation of public environments		
	Suspended all foreign travel requests from staff		
4. Educational Training for All Hospital staff, Volunteers, and Contractors	Proactively arranged educational training and audits on all hospital staff ensure workplace safety		This familiarises staff members with relevant safety and prevention strategies and helps them preserve a calm mood at work.
5. Construction of a Resilient Epidemic Prevention Network Within the Community	Connected with primary care clinics, nursing home facilities, borough chiefs, and pharmacies		Our hospital has constructed a community epidemic prevention networks for fighting COVID-19 epidemic.
	Provided relevant information and health care assistance		

### 1. Entry and exit access control

Access control limited entering and exiting of personnel. Person permitting to enter hospital was screened regarding body temperature, travel history, occupation, contact history, and cluster history [4] by designated professionals to rule out the high-risk. Efficiently, individuals’ travel history could be presented in their National Health Insurance cards (NHI IC cards) since 14 February 2020. Specially, inquired respiratory tract disease and requires all had to wear mask. This strategy could prevent cross-infection among persons and cut off potential transmission routes [5].

Record showed there were 25 677 persons entered hospital and received treatment during 14 February and 15 March 2020. A total of 324 persons (1.26%) had a travel history to epidemic affected places, 304 persons (1.18%) were required self-administered health management, 21 persons (0.08%) were home quarantined and 2 persons (0.01%) required mandatory quarantine. This strategy is usefully distinct from the high- and low-risk persons.

### 2. Management of wards

Since 1 March 2020, Taiwan started implementing hospital subdividing and triaging strategies, which included subdividing buildings, floors, wards, and units. Subdividing implies limiting the job descriptions of hospital staff, from cleaning personnel to health care personnel, which in turn reduces each person’s area of impact. Divided zones, divided times, and divided groups ensure that fixed personnel work in fixed zones during fixed shift periods, which prevents the staff from covering extensive areas during work and infecting each other, resulting nosocomial infections. It also protects the health care workforce from the scenario where too many health care personnel are required to isolate themselves. Triaging, in contrast, denotes making clear distinctions among patients based on their risks in aspects, such as entry and exit control, follow-up treatment, and inpatient zones. This strategy prevented health care persons from contacting with confirmed cases, or would result in short staffing and affecting the capacity of health care.

### 3. Management of hospital staff

According to Taiwan Centers for Disease Control (Taiwan CDC) record of 9 August, 2020, there are 480 confirmed cases and 388 of them (80.5%) being imported from outside Taiwan. Confirmed cases have already occurred in 187 countries throughout the world showing that foreign travel comes with a high risk of infection. Starting from 18 February, our hospital enforced measures of control on staff leaving the country. Anybody who wishes to travel to a Level Two (alert) country or region according to the Suggested Epidemic Levels for International Travel must first submit a request and self-isolate for 14 days upon their return. From 3 March, our hospital suspended all foreign travel requests from staff members.

All staff need to be on the roster lists. Their body temperature is measured daily, and self-report any respiratory tract-related symptoms. Moreover, the hospital distributed at least one mask for each staff each day, installed more hand-washing devices, and increased the sanitisation of public pathways and elevators [6].

It has been proven that asymptomatic carriers become latent transmitters, which makes the protection of hospital personnel even more essential [7,8]. Upholding the safety of health- care persons and providing them with a safe environment would protect them from the risk; that is crucial to ensure whose willingness to continue engaging in health care work.

### 4. Educational training for all hospital staff, volunteers, and contractors

Our hospital has proactively arranged various educational training courses for all hospital staff so that individuals can ensure workplace safety. The training courses include the following:

1. Carrying out educational training and audits on all hospital staff. Some of the content covered is as follows: demonstrations and practices on putting on and taking off personal protective gear, treatment of patients according to divided zones and patient transportation routes, bleach mixing, and reinforcement of hand hygiene.
2. Opening special courses to all health care staff in the hospital. Some of the content covered is as follows: diagnosis of COVID-19, patient-handling suggestions, control guidelines, and discussions on the treatment of severe cases.
3. Providing frontline health care staff with safety and protection training on intubation and equipment.

This familiarises staff members with relevant safety and prevention strategies and helps them preserve a calm mood at work rather than become concerned or anxious [9].

### 5. Construction of a resilient epidemic prevention network within the community

After the impact that SARS had on Taiwan’s public health and health care systems in 2003, a study revealed a severe lack of understanding among community members toward diseases and methods for preventing them, even to the point of demonstrating discriminatory and biased attitudes towards sick members of the public [10]. In the initial stage of the COVID-19 outbreak, Min-Sheng Community Hospital had already begun serving a core function in the community’s epidemic prevention system. The hospital connected with primary care clinics (32 clinics), nursing home facilities (24 facilities), and borough chiefs (26 people) at the earliest time possible and provided relevant information and health care assistance. This included the following:

1. Primary care clinics: Community nurse practitioners served as the sole window of contact, which allowed primary physicians to speedily arrange for presumptive patients to be transferred to our hospital, where further testing and isolated treatment took place.
2. Nursing home facilities: Designated staff from our hospital’s discharge planning centre served as the sole window of contact. Apart from offering epidemic consultation services, they promptly arranged for facility residents to receive isolated treatment in a hospital as soon as they showed any symptoms.
3. Members of the community: We established network contact with residents of the local boroughs. Aside from providing the latest information on the epidemic, we arranged educational courses on epidemic prevention.
4. Community pharmacies: Our hospital collaborated with pharmacies and adopted the method of releasing prescriptions to members of the public with chronic diseases and encouraging them to pick up their medication at pharmacies within their communities.

Our hospital has constructed a community epidemic prevention networks for fighting COVID-19 epidemic.

## CONCLUSION

Because of the experiences in fighting the SARS epidemic on 2003 and engaging in epidemic prevention for avian influenza and middle east respiratory syndrome that followed, Taiwan has acknowledged the crucial role that community hospitals play in community epidemic prevention networks. Efficient, well-trained community hospitals can sustain the operations of primary health care systems and alleviate large medical centres’ stress of receiving large waves of patients. This is because community hospitals can use their experience of being deeply engaged with their community to make the swiftest epidemic prevention management responses, preventing massive epidemic outbreaks in the community. Fighting the COVID-19 global pandemic is a serious challenge that has proven the effectiveness of the actions taken by Taiwanese community hospitals, including triaging patients visiting hospitals, subdividing health care teams, managing health care staff, and constructing community epidemic prevention networks. These strategies can serve as models for fighting biological disasters in the future and can act as references for community hospital health care systems.
